# Interprofessional Communication Team for Caregivers of Patients Hospitalized in the COVID-19 Wards: Results From an Italian Experience

**DOI:** 10.3389/fmed.2021.621725

**Published:** 2021-09-13

**Authors:** Sara Carletto, Michele Corezzi, Maria Francesca Furmenti, Elena Olivero, Paola Rapicavoli, Paola Rossello, Maria Rosa Stanizzo, Andrea Bovero

**Affiliations:** ^1^Department of Neuroscience “Rita Levi Montalcini”, University of Torino, Turin, Italy; ^2^Clinical Psychology Unit, University Hospital “Città della Salute e della Scienza di Torino”, Turin, Italy; ^3^Department of Public Health Sciences, University of Torino, Turin, Italy; ^4^Department of Quality and Safety Healthcare, University Hospital “Città della Salute e della Scienza di Torino”, Turin, Italy

**Keywords:** health communication, caregivers, COVID-19, psychological distress, pandemic

## Abstract

**Background:** During the COVID-19 pandemic, emergency restrictions did not allow clinician family meetings and relatives' visits. In Molinette Hospital, a new communication model between healthcare providers and families of COVID-19 affected patients was developed by a team of physicians and psychologists. The study's aims were to investigate caregivers' distress and to analyse their satisfaction with the communications provided.

**Methods:** A cross-sectional study was conducted among caregivers of patients of Molinette Hospital COVID wards. Between April and June 2020, all caregivers were contacted 2 weeks after the patient's discharge/death to assess their satisfaction with the communications received through an online survey.

**Results:** A total of 155 caregivers completed the survey. Caregivers' distress level was found to be higher in women than men (*p* = 0.048) and in caregivers whose relative died compared to the caregivers whose relative was discharged (*p* < 0.001). More than 85% of caregivers defined communication “excellent”/“very good”; being male was associated with higher satisfaction levels than women (β = −0.165, *p* = 0.046). Besides daily communication, 63 caregivers (40.6%) received additional support from a psychologist of the team.

**Conclusions:** To our knowledge, this is the first study presenting, in an emergency, a new model of communication provided by a team of physicians and psychologists, and analyzing satisfaction with it. This model was highly appreciated by caregivers and it limited the discomfort caused by the restrictions on relatives' visits. It would be interesting to further evaluate the possibility of extending a communication model that includes doctors and psychologists in routine clinical practice.

## Introduction

Communication between healthcare providers, patients, and families has been identified as the most important and least accomplished factor regarding quality of care in the subintensive and in the intensive care units (ICU) (1). It was found that effective communication with patients in the ICU improves clinical decision-making (1) and promotes family satisfaction as well as their psychological well-being (2, 3). Clinical practice guidelines for support of the patient and their family report a number of recommendations, including frequent communications and repeated meetings concerning the care of the patient to reduce family stress and to be consistent with communication (4). Several studies have highlighted that communication with the caregivers is one of the most highly valued aspects of care (5–7). The most important family concerns were: having timely information (8, 9), receiving honest information, and support, comfort, proximity, and reassurance (8, 10). Effective communication improves family satisfaction, trust in the ICU physicians, clinical decision-making, and psychological well-being of family members (8, 11).

From January 2020, the novel coronavirus SARS-CoV-2, and the consequent disease Covid-19, has spread all over the world, creating an extraordinary situation of sanitary emergency, evolving in the pandemic that the World Health Organization (WHO) declared on the 11th of March (12). Since the beginning, Italy has been one of the most affected European countries and the Italian government had to drastically introduce new societal rules and legal provisions, limiting population movements, and social life. The increasing number of infected people put a considerable pressure on the Italian National Health System at all levels; hospitals hardly managed the increasing number of infected people who needed different levels of care and, therefore, many extraordinary measures were established such as the prohibition of access to caregivers in hospitals, suspension of non-urgent health services and the improvement of remote working among employees. An important weakness of the health care system was the lack of availability of equipment required for the protection of patients and health care workers. Face masks, for example, were unavailable for many weeks. On one side it was important to reduce any unnecessary potential exposure to infection, but on the other the importance of communication with patients and with their caregivers was valued. Therefore, COVID-19 pandemic restrictions did not allow clinician family meetings and relatives' visits to their beloveds; due to these difficulties, communication with caregivers should be conducted in the most transparent and comprehensive manner, to avoid an increase in the anxiety that the family may experience during this time (13). Thus, to overcome these issues, the hospital adopted telephone communications to allow family members to receive news about their relatives' clinical updates and to contact them, when possible. Phone calls cannot substitute the real presence at the bedside, but they can represent an alternative model of communication between relatives. Clinicians reported that families, during their telephone conversations on COVID-19, experienced the psychological burden and distress of not being able to see and care for their loved ones. This distress also seems to be exacerbated by the lack of information about this disease and the restrictions put in place to prevent its spread (14, 15). Moreover, patients remain alone all day without support, except that provided by the health care workers. It is well-known that family support in a “Mediterranean” country is of paramount importance. Before this crisis, in the hospital care units, family members were allowed to stay alongside their loved ones. In the first 2 weeks after the restrictions enacted by the Health Minister, we have patients and family members been discouraged to do this, drastically changing our way of operating.

Another critical issue was the alternance of the different clinicians due to the work shift who gave information by phone to the patient's relatives. This could leave families that are already stressed by the isolation and the serious condition of their loved one, confused as to who is in charge and to whom they should ask their questions (16, 17). The pandemic has created a public health emergency that is still altering the provision of health care services and affecting the quality and safety of health services. The above-described situation was daily experienced both in COVID and no-COVID wards of our health organization, the Molinette Hospital, inside the University Hospital of “Città della Salute e della Scienza di Torino.” The Molinette Hospital is a general, third-level hospital with nine hundred beds, located in Piedmont region, Italy, one of the most affected areas during the pandemic.

At the beginning of the SARS-CoV2 pandemic, the aim of the Health Management of Molinette Hospital and of the Quality and Safety Healthcare Department was to provide clear and consistent communication to the patient's relatives, and therefore decided to begin an experimental project to improve the quality and outcomes of the communication between healthcare providers and families of COVID-19 affected patients, hospitalized in the COVID wards and in the sub-intensive care. Despite a number of resources or guidance proposed by healthcare professionals (18), neither evidence- nor consensus-based guidelines about COVID-19 communication in hospitals and in palliative care in COVID-19 were available. Furthermore, a survey among hospices in Italy revealed that healthcare professionals lacked a communication guideline on care for people dying from COVID-19 (19). The new communication model was developed in accordance with the principles of humanization of clinical care (20), and carried out by a team composed by physicians from the Health Management and psychologists from the Clinical Psychology Unit. The team gave medical information to the families, including daily clinical updates (every day in the early afternoon) through phone calls, accurate but also comprehensible for the relatives (21). At the beginning of each phone call, all relatives were informed of the presence, during the conversation, of a physician and of a psychologist and of the possibility of receiving psychological support by a psychologist of the team. The model was implemented in COVID wards with low intensity of care (not ventilated patients) and subintensive COVID wards (Continuous Positive Airway Pressure and non-invasive ventilated patients); the medical and nurse teams of these wards were heterogeneous and usually not specifically trained in giving information to families and caregivers in emergency situations.

To our knowledge, this is the first collaboration between physicians and psychologists during the pandemic, that provided patients clinical and emotional information about the coronavirus and at the same time faced problems related to the distress and suffering of the relatives.

Therefore, this was a hypothesis-generating study with the aims to analyse caregivers' satisfaction with phone communications provided by a team of physicians and psychologists and to investigate caregivers' distress (anxiety and depression), their emotional experiences and their perception of the adequacy of social support during the COVID emergency.

## Materials and Methods

### Design and Participants

This study is a cross-sectional study including caregivers of hospitalized patients in the COVID wards and subintensive care units of the Molinette Hospital. All caregivers were contacted by phone 2 weeks after the patient's discharge or death to ask for their email address by a psychologist from the Hospital's Clinical Psychology Unit, who was not involved in the communication team, to assess their satisfaction with the daily conference calls. An email with the link to the survey, created with Google Form, was sent to those who agreed to participate. All questions in the survey were mandatory to avoid missing data. Raosoft® was used to determine the minimum sample size of 150, based on a 5% margin of error, 95% confidence level, 50% response distribution, and a population of 245 (total of caregivers of patients hospitalized in our hospital COVID-19 wards between March and May 2020). Data were collected between April and June 2020. The study was approved by the local Research Ethics Committee (no.39960/2020) and written informed consent was obtained from all participants. The reporting of this study conforms to the Strengthening the Reporting of Observational Studies in Epidemiology (STROBE) statement (22).

### Communication Team Model

Basing on a theoretical and practical analysis of the emergency context, healthcare workers involved in the first line during the emergency period faced long and lengthy work shifts, stressful from a physical and psychological point of view (23, 24). To partially relieve first-line workers, a mixed team composed by medical doctors and psychologists was built by the Health Management and Quality and Safety Healthcare Department; this team, hereafter COVID Communication Team (CCT), aimed to communicate with COVID-19 patients' relatives, to give them a sense of certainty and safety and clear, detailed and daily information on the condition of their loved ones, to allow them to re-elaborate the experience of suffering.

The CCT received daily information in a secure and telematics way (corporate encrypted email) from the ward's doctors after their daily briefing; all reported information shall be communicated to family members through a standardized format personalized by clinicians with patient-specific information. This format contains information about the patient's cognitive state, clinical parameters, ongoing therapy, and clinical activities that will be undertaken in the following days. CCT collectively analyzed and discussed the documents and called the designated family member during the afternoon. During the first interview, a medical doctor (MD) in charge of the team introduced the communication service and explained to the family member what mediated communication means and how the psychological support works. After the introduction phase, the CCT MD communicated the clinical information, taking notes on a special register for the specific requests of the caregivers. These notes were then sent via e-mail to the COVID wards' clinicians, thus ensuring a continuity of communication both within the CCT members and with the COVID wards healthcare staff. Telephone interviews were carried out every day in the same way, from Monday to Sunday. When the patient was in good clinical condition, caregivers could also ask CCT for a direct video call with their relatives, which was made possible by using the tablet computers available in all COVID wards with the help of the healthcare workers.

Moreover, the CCT MD communicated to relatives, in accordance with the medical and nursing staff of the COVID wards, a possible death of the patient in the hospital, supported by the clinical psychologist. Telephone interviews also allowed CCT psychologists to deal with family members with problems related to their own suffering, generated by social isolation, loneliness, and stress inherent to an emerging situation. For these reasons, it was useful to offer a telephone psychological support service for all family members when requested in a dedicated moment at the end of the daily CCT phone calls.

### Study Measures

The online questionnaire was anonymous and took about 15 minutes to be completed. The survey was composed of three sections: in the first section socio-demographic data of the caregiver (age, gender, marital status, level of education, and profession) and data on the clinical course of patients (discharged/transferred or deceased) were collected. In the second section, three validated tools were administered to assess caregivers' emotional distress, perceived social support, and satisfaction with the communication received by the doctor-psychologist team. Specifically, the following questionnaires were administered:

- the Multidimensional Scale of Perceived Social Support (MSPSS) (25, 26), a self-report 12-item scale designed to measure perceived social support. The total score range is 1–7, with higher scores indicating greater perceived support.

- the Distress Thermometer (DT), derived from the Distress Thermometer developed by the National Comprehensive Cancer Network (27). It is a one-item, 11-point Likert scale that ranges from zero (no distress) to ten (extreme distress), with which caregivers indicate their level of distress in the past 7 days.

- a set of questions regarding the satisfaction with the communication received by the healthcare team, taken from Supportive Care Need Survey-short form (SCNS-SF 34) (28, 29), a rating scale designed to assess unmet needs in cancer patients. The distribution of answers was on a 5-point Likert scale, which could vary from “poor” to “excellent,” or from “not at all satisfied” to “extremely satisfied” or from “not at all” to “very much,” depending on the type of question.

Finally, in the third section, two open-ended questions were asked to collect criticism and possible suggestions to improve the communication service.

### Statistical Analysis

Data were processed and analyzed using the Statistical Package for Social Sciences (SPSS version 22.0; Chicago, IL, USA). Percentage values, means and standard deviations (SD) were used to describe the sample. Differences between groups were calculated using Mann-Whitney *U*-test for continuous measures. A multivariable linear regression analysis was performed to determine the association between gender, age, distress level, MSPSS score, and patient clinical course as independent variables and overall satisfaction score as the dependent variable. All tests were two-sided. A *p* < 0.05 was considered statistically significant.

## Results

A total of 155 caregivers completed the online survey (response rate: 155/245 = 63%). Of these, 71% were female and 29% were male. The mean age was 53.8 (SD = 12.94), with a range between 21 and 85 years. The other socio-demographic characteristics of caregivers and their relative clinical data are detailed in Table 1.

**Table 1:** Socio-demographic data of caregivers and clinical course of their relatives (*N* = 155).

**Variable**	***n* (%)**
**Level of education**	
Primary school	2 (1.3)
Low secondary school	39 (25.2)
High secondary school	63 (40.6)
University	51 (32.9)
**Marital status**	
Single	30 (19.4)
Married/cohabitant	98 (63.2)
Separated/divorced	12 (7.7)
Widowed	15 (9.7)
**Employment status**	
Unemployed	17 (11.0)
Employed	101 (65.2)
Retired	35 (22.6)
Student	2 (1.3)
**Living condition**	
Alone	28 (18.1)
With spouse	42 (27.1)
With spouse and children	69 (44.5)
With children	9 (5.8)
With parents	7 (4.5)
**Degree of kinship with the patient**	
Spouse/cohabitant	59 (38.1)
Parent	41 (26.5)
Son/daughter	23 (14.8)
Grandparent	1 (0.6)
Sibling	11 (7.1)
Other relative	13 (8.4)
Friend	7 (4.5)
**Duration of patient hospitalization**	
<7 days	31 (20.0)
Between 8 and 14 days	51 (32.9)
Between 15 and 20 days	27 (17.4)
More than 21 days	46 (29.7)
**Clinical status of patient**	
Discharged	112 (72.3)
Deceased	43 (27.7)

Caregivers reported a distress mean score of 6.59 (SD = 2.88) and a MSPSS mean score of 5.96 (SD = 1.18). The distress level was found to be higher in women than men (*p* = 0.048), and in caregivers whose relative died compared to the caregivers whose relative was discharged from the hospital (*p* < 0.001). No significant differences were found for other variables.

Table 2 describes caregivers' satisfaction with the communication service received by the healthcare team. Possible answers ranged on a 5-point Likert scale. Question number ten, “Did they encourage you to ask questions?”, obtained the lowest score (mean=3.83, SD=1.22), while question number seven, “Did they give you all the information you needed?,” had the highest score (mean = 4.84, SD = 0.87).

**Table 2:** Caregivers' satisfaction with the communication service received by the healthcare team (*N* = 155).

**Questions**	**Mean (SD)^a^**
1) Overall, how do you rate the service received?^b^	4.44 (0.89)
2) How satisfied are you with having received daily updates regarding the clinical situation of your relative?^c^	4.59 (0.84)
3) How welcomed you felt by the health team?^d^	4.37 (1.03)
4) Did you feel treated with respect?^d^	4.73 (0.60)
5) Did they understand your main concerns?^d^	4.52 (0.87)
6) Did they let you speak without interrupting?^d^	4.59 (0.80)
7) Did they give you all the information you needed?^d^	4.84 (0.87)
8) Did they use words that were easy for you to understand?^d^	4.58 (0.83)
9) Did they verify that you understood everything?^d^	4.47 (0.94)
10) Did they encourage you to ask questions?^d^	3.83 (1.22)
11) Did they communicate the progress of your relative's treatment?^d^	4.54 (0.089)
12) Did they show attention and interest?^d^	4.52 (0.82)
13) Did they give you the right time?^d^	4.43 (0.93)

a *SD, Standard Deviation*.

b *distribution of answers was on a 5-point Likert scale from “poor” to “excellent”*.

c *distribution of answers was on a 5-point Likert scale from “not at all satisfied” to “extremely satisfied”*.

d *distribution of answers was on a 5-point Likert scale from “not at all” to “very much”*.

Overall, the daily communication service received was rated as excellent by 63.2% (*n* = 98), as very good by 23.2% (*n* = 36), as good by 9.7% (*n* = 15), as fair by 1.9% (*n* = 3), and as poor by 1.9% (*n* = 3) of the caregivers.

In addition to daily communication with the healthcare team, 63 caregivers (63/155; 40.6%) received additional support from a psychologist of the team. The satisfaction scores regarding this support are described in Table 3.

**Table 3:** Caregivers' satisfaction with the support received by psychologists of the healthcare team (*N* = 63).

**Questions**	**Mean (SD)^a^**
1) How satisfied were you with the communication with the psychologist?^b^	4.37 (0.90)
2) How much did you feel encouraged and supported by the psychologist?^c^	4.24 (0.93)
3) How much support did you feel in dealing with that difficult situation?^c^	4.13 (1.13)

a *SD, Standard Deviation*.

b *distribution of answers was on a 5-point Likert scale from “not at all satisfied” to “extremely satisfied”*.

c *distribution of answers was on a 5-point Likert scale from “not at all” to “very much”*.

The multiple regression model showed that only gender had a significant predictive value for the overall satisfaction score [β = −0.165, *p* = 0.046; Model adjusted *R*^2^ = 0.027, *F*_(5, 148)_ = 1.84, *p* = 0.109], with being male associated with higher satisfaction levels. Age, distress level, perceived social support, and discharge/death of the caregiver's relative had no predictive value on the caregivers' overall satisfaction score with the communication received.

Among the 155 caregivers, only 29 reported some critical issues. The following were the main ones: seven people (24.1%) would have preferred to receive the clinical updates regarding their relative's health status directly from the ward doctors and not only from the CCT. Six caregivers (20.7%) reported that they had received too little information, and five (17.2%) stated that they have received little attention and support after the discharge from the hospital of their relatives, also due to poor information received from the primary care health services.

Finally, 24 caregivers made suggestions to improve the service: six people (25%) suggested to develop a way to communicate directly with ward doctors, four (16.7%) suggested to receive information more than once a day, and three caregivers (12.5%) suggested to increase the level of empathy during phone-calls made by the communication team.

## Discussion

Communication with caregivers is one of the most highly valued aspects of care (5–7), especially in a health emergency such as COVID-19 pandemic (30, 31). This study aimed at presenting a new model of communication between healthcare providers and families of COVID patients provided by CCT, a team of physician and psychologists, and investigating the caregivers' satisfaction with it. In addition, it investigated caregivers' distress and their perception of the psychological support received during the COVID emergency.

The satisfaction score is high, as more than 85% of respondents defined the communication “excellent” or “very good.” Moreover, the satisfaction level does not appear to be associated with the clinical status of the patient (discharged or deceased). This could be indicative of good bereavement care. According to Morris and collaborators, it is crucial for hospitals to adopt a proactive stance during a public health emergency, to offer universal bereavement care to all families: in the context of the COVID-19 pandemic there is a high possibility that patients will die alone, separated from their loved ones and therefore, there is a sense of urgency for institutions to provide support to bereaved family members (30). To do this, CCT made bereavement calls in the first week after the patient's death, that may have helped grieving families to know that the patient and family were remembered, contributing to the quality of the end-of-life care. Despite this, as expected, the distress level was found to be significantly higher in caregivers whose relative died compared to caregivers whose relative was discharged from the hospital.

Gender was found to be related both to distress and satisfaction levels. Women resulted having higher distress and lower satisfaction levels with the communication service compared to men. This result is in line with the current literature, as higher distress in women was found also in the general population in other studies regarding the COVID pandemic (32, 33).

According to the Italian Istituto Superiore di Sanità report (34), caregivers preferred video calls to communicate with hospitalized patients and with healthcare personnel, because of the possibility of characterizing the face and the aspects of healthcare personnel and to establish a closer relationship (35). Interestingly, none of the caregivers of our sample reported this request. In addition, not all caregivers had the possibility to make video calls. Instead, direct communication between patients and caregivers was encouraged through video calls where possible, or via traditional telephones, with the help of health personnel for patients who needed it. Indeed, recent literature highlights the importance of facilitating communication between patients and family members (30).

Communication between staff and caregivers occurred every day, at about the same time, by the same communication team. This may have contributed to a good level of satisfaction, since family members of a critically ill patient appreciate proactive and regular communication (36), meanwhile frequent communications and repeated meetings concerning the care of the patient could reduce family stress (4). Other studies also highlighted the importance of receiving timely information (8, 10). To confirm this, in our study the satisfaction level of the daily update frequency was very high. Therefore, clear, specific, and detailed information decreased the feeling of insecurity, doubt, and fears of relatives and gave them the opportunity to imagine the situation of the beloved. In addition, good communication reassured families about the optimal care of the patient at a time when trust in a highly pressured health system was often questioned.

Few caregivers would have preferred to receive information several times a day. To analyse this result, it is important to contextualize the study in the emergency period: the request to receive information more than once a day probably also derives from the fact that family members were often all day alone at home, unable to go out to see relatives and friends, with low perceived social support (37). Some authors have recommended contact twice a day if the patient is at imminent risk of life or is dying (38). However, it should be noted that 40% of the caregivers who responded to the survey received additional support from the team of psychologists, with a good level of satisfaction. This additional support was also considered important in previous studies (34, 39).

Satisfaction about the completeness of the information received was highly valued, with only a few caregivers complaining about having received too little information. This may reflect the validity of the communication format between CCT and healthcare professionals in wards, with patients clinical and emotional condition. Completeness of information is an important point of communication; indeed, information should be given in a comprehensive and transparent manner to reduce the caregiver's anxiety (13). On the contrary, some caregivers felt they were not encouraged enough to ask questions. During the peak period of the pandemic, around 70–80 caregivers were called per day, therefore it is possible that despite the CCT willingness to dedicate sufficient attention to all caregivers there was not enough time to address all their questions. This aspect would need further investigation to better understand the specific needs of caregivers.

Some caregivers reported a lack of continuity in support once the patient was discharged from the hospital. This aspect, combined with the fragmented scenario of the emergency period when each care structure adopted its own communication strategy, is a critical point that must necessarily be further addressed. Communication with patients and their relatives is a major issue in medicine and the experience of the COVID-19 pandemic could be a starting point for a common model, adaptable to different health settings. The design and implementation of our communication model was possible also thanks to the human resources made available by the reduction of the workload in non-COVID hospital wards and outpatient services (36).

This study presents several strengths. To our knowledge, this is the first study evaluating the satisfaction of a new model of communication for emergency situations provided by a collaborative team of physicians and psychologists. Moreover, the size of the sample was high, and it was conducted in the biggest hospital in the Italian region most affected by COVID-19 at this time.

However, the present study has some limits that should be acknowledged. First of all, the lack of data on non-responders may have led to possible bias. Responder caregivers could be more satisfied with the communication service than non-responders. This limitation should be taken into account in the interpretation and generalizability of findings (40). Moreover, using an online questionnaire may have limited participation, excluding older caregivers who are less prone to the use of technological tools (41). On the other hand, an online survey was considered the most suitable tool to guarantee the anonymity of participants (42). Moreover, the satisfaction toward the communication model of the clinicians working in the COVID wards was not investigated. The lack of direct interaction with patients' caregivers could have been not easily accepted by all healthcare workers, despite the reduction of the workload.

In conclusion, the communication model presented so far was highly appreciated by caregivers and it limited the distress related to the restrictions imposed on the hospital ward visits. It would be interesting to further evaluate the possibility of extending a communication model that includes a doctor and psychologist even outside the pandemic, in routine clinical practice.

## Data Availability Statement

The data that support the findings of this study are available on request from the corresponding author.

## Ethics Statement

The study was conducted in accordance with the Declaration of Helsinki, and was approved by the Ethical Committee of A.O.U. Città della Salute e della Scienza di Torino, Italy. Written informed consent was obtained from all participants.

## Author Contributions

AB and SC conceived the study idea. MC, PRa, PRo, and MS performed the data collection. SC performed the statistical analyses. AB, SC, MF, and EO drafted the first version of the manuscript. All authors contributed to the study design, have discussed the results and revised this manuscript critically for important intellectual content, read, and approved the final version.

## Conflict of Interest

The authors declare that the research was conducted in the absence of any commercial or financial relationships that could be construed as a potential conflict of interest.

## Publisher's Note

All claims expressed in this article are solely those of the authors and do not necessarily represent those of their affiliated organizations, or those of the publisher, the editors and the reviewers. Any product that may be evaluated in this article, or claim that may be made by its manufacturer, is not guaranteed or endorsed by the publisher.
