# Supplementary material for: Interprofessional Communication Team for Caregivers of Patients Hospitalized in the COVID-19 Wards: Results From an Italian Experience
Source: Front Med (Lausanne). 2021 Sep 13;8:621725. doi: 10.3389/fmed.2021.621725 (PMC8473685; doi:10.3389/fmed.2021.621725)
Supplement: Supplementary file 1 [file Table_1.PDF]

Supplementary file

Carletto et al., "Interprofessional communication team for caregivers of patients hospitalized in the COVID-19 wards: results from an Italian experience."

CCT STANDARDIZED FORMAT FOR CLINICAL DATA

WARD NAME
DATA

BED NUMBER, NAME and SURNAME of the patient, AGE
DATA OF THE HOSPITALIZATION

ADMISSION DIAGNOSIS
OXIGEN SUPPLMENT: yes/no; amount
COVID-19 SWAB: data and results

PAST MEDICAL HISTORY
HOME THERAPY

RECENT MEDICAL HISTORY

TODAY CLINICAL CONDITION
CLINAL PROGRAM: medical investigations, medical advices

RELATIVE CONTACTS: number and relationship
FEEDBACK: feedback after today phone call to the relative
MEDICAL DOCTOR: name of the medical doctor of the communication team who did the phone call
FURTHER PSYCOLOGICAL ADVICE REQUESTED: yes/no, name of the psychologist

CCT OUTLINE OF PHONE COMMUNICATION WITH THE CAREGIVER

1. PRESENT YOURSELF

- First time you speak to the caregiver, present yourself (name, surname, role), the communication team (composed of a physician and a psychologist) and specify that the communication team is going to call him/her every day, at the same hour, during the hospitalization of their relative.

2. FIND OUT ABOUT THE PERSON YOU ARE TALKING TO

- Tell him/her the name of the person you are talking/calling about and ask their relationship with that person (e.g., wife/husband; daughter/son; parent; sister/brother).
- Ask the relative about his/her current health status.
- Find out what the person you are talking to already knows and/or expects, and how he/she feels about that.
- Listen for what he/she understands; for worries and concerns; for gaps in his/her understanding.

3. BRING THE PERSON TOWARDS AN UNDERSTANDING OF THE SITUATION

- Summarise the new developments, check if him/her understands what you mean if you use medical words.
- Tell him/her clearly what you know and/or expect to happen, accordingly to the notes written by the ward doctor.
- Check if him/her understands the clinical situation and encourage him/her to ask if he/she has any doubts or questions.
- Ask him/her if he/she would like to receive further psychological advice.

4. CLOSE THE PHONE CALL

- Ask the relative if he/she has any messages or requests about his/her relative.
- Remind him/her that he/she will receive another call from the CCT the following day.
- Write down a short summary of the phone call and send it to the ward doctors.
